# Real-world hepatitis C treatment outcomes and reinfections among people who inject drugs at a needle and syringe program in Stockholm, Sweden

**DOI:** 10.1186/s12954-023-00801-1

**Published:** 2023-06-12

**Authors:** K. Lindqvist, Z. Thorin, M. Kåberg

**Affiliations:** 1 Stockholm Centre for Dependency Disorders, Stockholm Needle Syringe Program, Stockholm, Sweden; 2 Department of Global Public Health, Karolinska Institutet, Sprututbytet, S:t Görans Sjukhus, Akutvägen 29, 112 81 Stockholm, Sweden

**Keywords:** Hepatitis C, People who inject drugs, Needle and syringe program, Stimulant use, Hepatitis C reinfection

## Abstract

**Background:**

People who inject drugs (PWID) represent a population with an increased prevalence of hepatitis C (HCV) infections. HCV treatment among PWID is essential to reach the WHO goal of eliminating HCV as a major public health threat by 2030. Despite better understanding of PWID subgroups and changes in risk behaviors over time, more knowledge about HCV treatment outcomes in different HCV prevalence populations and settings is warranted to enhance the continuum of care.

**Methods:**

All Stockholm Needle and Syringe Program (NSP) participants who initiated HCV treatment between October 2017 and June 2020 were HCV RNA tested at end of treatment and twelve weeks thereafter to confirm cure with a sustained virological response (SVR). All cured participants were prospectively followed from SVR to the last negative HCV RNA test or a subsequent reinfection, until October 31, 2021.

**Results:**

Overall, 409 NSP participants initiated HCV treatment, 162 at the NSP and 247 in another treatment setting. There were a total of 6.4% treatment dropouts (*n* = 26), 11.7% among participants treated at the NSP and 2.8% among those treated elsewhere (*p* < 0.001). Stimulant use (*p* < 0.05) and not being in an opioid agonist treatment program (*p* < 0.05) was associated with dropout. More participants treated outside the NSP were lost to follow-up between end of treatment and SVR (*p* < 0.05). During follow-up post-SVR, 43 reinfections occurred, corresponding to a reinfection rate of 9.3/100 PY (95% CI 7.0, 12.3). Factors associated with reinfection were younger age (*p* < 0.001), treatment while in prison (*p* < 0.01) and homelessness (*p* < 0.05).

**Discussion:**

In this high HCV prevalence NSP setting, with a majority of stimulant users, treatment success was high and the level of reinfections manageable. To reach HCV elimination, there is a need to target specific PWID subgroups for HCV treatment, in both harm reduction and adjacent healthcare settings frequented by PWID.

## Introduction

An estimated 57 million people worldwide are infected with hepatitis C virus (HCV) [1]. Among people who inject drugs (PWID), the HCV prevalence is high and the major route for HCV transmission is sharing of unsterile injection equipment (needles/syringes, cookers and filters) [2, 3]. Among the 15.6 million people worldwide with recent injection drug use (IDU), 6.1 million (39%) are estimated to be HCV infected [4]. In 2016, the World Health Organization (WHO) presented a strategy to eliminate hepatitis B (HBV) and HCV as a major public health threat in the world by 2030 [5]. The HCV elimination targets include a 90% reduction of new cases, 80% of cases treated and a 65% reduction in HCV-related deaths [6]. With current HCV direct-acting antiviral (DAA) treatment, cure rates > 95% can be achieved with 8–12 weeks of treatment [7]. International guidelines recommend HCV treatment among PWID as a prioritized task, to reduce risk of continued transmission [8–10]. However, even though treatment as prevention is a proven effective strategy [11, 12], success in HCV elimination among PWID also relies on high coverage of evidence-based harm reduction services like needle and syringe programs (NSP) and opioid agonist treatment (OAT) [13, 14].

To increase HCV treatment among PWID, continued scale-up of harm reduction programs is needed with an ambition to facilitate HCV diagnosis and linkage to care [15], combined with further understanding of PWID subgroups, to bridge knowledge gaps regarding HCV treatment barriers [16]. Research shows that NSP significantly impacts both injection risk behavior and HCV transmission among PWID, particularly when combined with OAT [17–20]. Further, a growing body of literature suggests feasibility of HCV treatment among PWID and people on OAT regarding both adherence and treatment outcomes, i.e., sustained virological response (SVR) [21, 22]. However, some healthcare providers still raise concerns regarding compliance and adherence to DAA treatment in PWID, and challenges remain in countries still facing DAA reimbursement restrictions [23–25]. As a result, studies from different countries suggest that HCV treatment uptake in PWID globally is low, limited to 1–2% of the population [26–28].

Among HCV-treated PWID, a somewhat lower noted SVR rates in treatment studies could be explained by PWID being lost to follow-up (LTFU), rather than treatment failure. However, research also shows that LTFU has been associated with recent IDU risk behaviors, suggesting higher risk of HCV reinfection [29, 30].

In 2019, a report on Sweden’s HCV elimination goals for 2030 was released, pointing to several challenges and knowledge gaps [31]. The report specifically underlined need for HCV treatment scale-up and to reach and retain PWID and subgroups in the continuum of care, i.e., a set of bundled healthcare and harm reduction interventions, and a need for more efficient collaboration between NSP and clinics offering OAT. Growing research on PWID in the Stockholm NSP has found a 55% HCV baseline prevalence and despite significant reduction in injection risk behaviors over time, a high HCV incidence rate around 22/100 person-years (PY) [32, 33].

Treatment uptake of HCV in active PWID has historically been low in Sweden and treatment in the OAT population also limited. Previous data, before the DAA era, showed that the overall lifetime uptake of HCV treatment among PWID on OAT was between 1 and 6% [34–36]. However, an observational study with data from the Swedish Prescribed Drug Registers noted an estimated cumulative DAA treatment uptake of 28% among OAT participants between 2014 and 2017 [37].

High HCV prevalence, incidence and injection risk behavior among PWID are strong indicators for prompt scale-up of HCV treatment to reduce overall prevalence [38, 39]. In 2018, all HCV reimbursement restrictions in Sweden were omitted giving universal access to treatment, and in 2020 acute HCV was included in Swedish HCV treatment guidelines [40]. HCV treatment was first introduced at the Stockholm NSP in late 2017 and expanded from 2018.

Despite better understanding of PWID subgroup risk behaviors and change over time, more knowledge about HCV treatment and outcomes in different HCV prevalence populations and settings is warranted to enhance the continuum of care. In this study, we use real-world HCV treatment data from the Stockholm NSP, a setting with a high HCV baseline prevalence and with a majority of stimulant users, to investigate HCV treatment outcomes for participants at the NSP. We aim to investigate socio-demographic and IDU-related differences between NSP participants receiving HCV treatment on site and those who received HCV treatment elsewhere (i.e., outside of the NSP). We also investigate HCV reinfection rates post-SVR among all HCV-treated NSP participants.

## Methods

### Participants and setting

The Stockholm NSP has previously been described in detail [30, 32, 33, 41]. Briefly, the Stockholm NSP is a multi-center (two fixed sites and one mobile unit) low-threshold program with a multidisciplinary care approach and staffed by physicians, counselors, midwifes and nurses specialized in infectious diseases and substance use. The NSP offers sterile needles/syringes, cookers and filters, infectious disease treatment, overdose prevention, contraceptive counseling and other midwife services, referrals to social services and substance use clinics and outreach activities. At enrollment, all participants answer 34 questions on socio-demographic and drug-related information. All participants are tested for HIV, HBV and HCV. Follow-up interviews and HIV and hepatitis testing are repeated every three to six months, and all data are entered into an electronic quality register database, InfCare NSP. The Stockholm NSP has since the start in 2013 enrolled over 4,400 participants. Yearly, approximately 1800 clients make 20,000 client visits.

### Fibrosis evaluation and HCV treatment at the NSP

In this study, all NSP participants that initiated HCV treatment between October 2017 and May 2020 were included. During the study period all participants with HCV were informed about access to HCV treatment through personal information from the staff and digitally through an information screen in the waiting rooms. HCV treatment was mainly nurse-led (i.e., nurses tested for HCV, including liver function tests and genotypes, performed liver stiffness measurement (LSM) with FibroScan, suggested DAA treatment strategy and monitored participant during and after treatment). LSM cutoffs were 7 kPa and ≥ 12.5 kPa for significant liver fibrosis and cirrhosis, respectively [42]. In cases where FibroScan was not accessible, the algorithm; APRI score < 1.0 in combination with < 15 years duration of IDU and an age < 35 years was used to exclude advanced fibrosis/cirrhosis [41].

A physician at the NSP confirmed initial treatment strategy, prescribed DAA and evaluated complicated treatment-related issues, when needed. All HCV diagnosis, evaluation, treatment and follow-up at the NSP were performed in accordance with Swedish HCV guidelines [43]. In most cases, DAA was dispensed on a weekly basis but also during longer intervals on-demand, if a participant was temporarily accommodated at a treatment home or while incarcerated. SVR, with a negative HCV RNA 12 weeks after end of treatment (EOT), defined successful treatment.

### NSP participants HCV treated outside the NSP

During the study period (October 2017 and May 2020), we also identified NSP participants who were HCV treated outside the NSP. These participants were identified through self-reported HCV treatment or were actively asked about HCV treatment by NSP staff and had a confirmed positive HCV RNA pre-treatment. Duration of treatment was confirmed through external medical charts and was registered in InfCare NSP. The majority of participants treated outside the NSP were treated at infectious diseases (ID) clinics, at OAT clinics and to a lesser extent in prison. For this study, there were no available data on pre-treatment evaluation, HCV genotype, level of fibrosis or choice of DAA among HCV treated outside the NSP. As a result, comparative analyses between participants treated at the NSP and outside the NSP were focused on demographic data, treatment outcomes and reinfection data.

### Follow-up and reinfection post-treatment

All successfully HCV-treated participants were followed from SVR to the last negative HCV RNA test or a subsequent reinfection, until October 31, 2021. With this follow-up time, all participants were followed up for a minimum of 12 months post-SVR. Reinfection was defined as a negative HCV RNA at SVR followed by a positive HCV RNA during follow‐up.

### Statistical analyses

Based on our previous research [30, 32, 33, 41, 44], nine sociodemographic-, drug- and HCV-related determinants were selected (described in detail in Table 1) for analyses. Demographic data are presented as proportions, mean or median levels with ranges and interquartile ranges (IQR). If more than two responses, all other responses were combined to make a dichotomized comparison category. The Chi-square test or Fisher exact two-tailed test was used to test categorical variables and the Wilcoxon rank sum test for continuous values. A *p* value of < 0.05 was considered statistically significant. Reinfection rates were defined as number of reinfections (*n* = *x*) per 100 person‐years (*x*/100 PY). Data were analyzed using JMP®, Version 15, SAS Institute Inc., Cary, NC.

**Table 1:** Characteristics of HCV-treated NSP participants at NSP and outside of the NSP (*n* = 409)

	All HCV treated (*n* = 409)	HCV treated at the NSP (*n* = 162)	HCV treated outside of the NSP (*n* = 247)	*p* value treated at NSP/outside of NSP
	*n* (%)	*n* (%)	*n* (%)	
Gender	*n* = 409	*n* = 162	*n* = 247	
Men	318 (77.8)	122 (75.3)	196 (79.4)	0.40
Women	91 (22.2)	40 (24.7)	51 (20.6)	
Age	*n* = 409	*n* = 162	*n* = 247	
Mean (SD)	44.8 (11.1)	46.8 (10.6)	43.6 (11.3)	< 0.01
Median (range)	45.5 (21–68)	49.0 (22–66)	42 (21–68)	
Country of birth	*n* = 401	*n* = 162	*n* = 239	
Sweden	346 (86.3)	139 (85.8)	207 (86.6)	0.88
Outside of Sweden	55 (13.7)	23 (14.2)	32 (13.4)	
Living situation	*n* = 339	*n* = 149	*n* = 190	
House/apartment	202 (59.6)	81 (54.4)	121 (63.7)	0.09
Support housing	65 (19.2)	31 (20.8)	34 (17.9)	0.58
Treatment home	27 (8.0)	10 (6.7)	17 (8.9)	0.54
Prison	5 (1.5)	3 (2.0)	2 (1.1)	0.66
Homeless	40 (11.8)	24 (16.1)	16 (8.4)	< 0.05
Predominantly drug injected	*n* = 408	*n* = 162	*n* = 246	
Stimulants/CS	230 (56.2)	123 (75.9)	107 (43.4)	< 0.0001
Opioids/OP	155 (37.9)	32 (19.8)	123 (49.8)	< 0.0001
Mixed CS/OP	22 (5.5)	7 (4.3)	13 (6.1)	0.82
Other drugs	1 (0)	0	1 (0)	–
Duration of IDU	*n* = 402	*n* = 160	*n* = 242	
Mean (SD)	22.5 (13.1)	23.3 (13.2)	22.0 (13.0)	0.33
Median (range)	22 (1–52)	23.7 (1.5–51.7)	21 (1–52)	
OAT	*n* = 409	*n* = 162	*n* = 247	
Yes	126 (30.2)	14 (8.6)	112 (45.3)	< 0.0001
No	283 (69.1)	148 (91.4)	135 (54.7)	
HIV	*n* = 409	*n* = 162	*n* = 247	
Positive	27 (6.6)	1 (0.6)	26 (10.5)	< 0.0001
Negative	382 (93.4)	161 (99.4)	221 (89.5)	
HBV	*n* = 408	*n* = 162	*n* = 246	
HBsAg positive	0 (0)	0 (0)	0 (0)	–
Seronegative	30 (7.3)	12 (7.4)	18 (7.3)	1.00
Immune (post-infection)	192 (46.9)	76 (47.1)	116 (47.2)	1.00
Vaccinated	186 (45.5)	74 (45.7)	112 (45.5)	1.00

## Results

### Participant characteristics

A total of 409 participants were included in the final analyses, of which 162 were treated at the NSP and 247 treated outside the NSP (Table 1). In 2017, 11 treatments were initiated among NSP participants, which increased to 146 and 203 for 2018 and 2019, respectively. During the first five month in 2020, treatment initiations decreased substantially, related to the COVID-19 pandemic.

Overall, 77.8% of participants were men and the mean age was 44.8 years. Age was higher among those treated at the NSP compared to those treated outside the NSP, mean age 46.8 years versus (vs.) 43.6 years (*p* < 0.001). Over half of the participants (56.2%) reported stimulants (amphetamine, cocaine, methylphenidate) as the most frequently used drug, with amphetamine as the predominant drug (91.3%). The second most common drugs used were opioids (37.9%), with heroin as the predominant drug (93.5%). Stimulants was more frequently used among participants treated for HCV at the NSP compared to those treated outside the NSP, 75.9% vs. 43.4% (*p* < 0.0001). Consequently, participants treated at the NSP reported less use of opioids, 19.8% vs. 49.8% (*p* < 0.0001) and OAT was more prevalent among those treated outside the NSP 45.3% vs. 8.6% (*p* < 0.0001). Furthermore, participants with a concomitant HIV infection received HCV treatment to a greater extent outside the NSP, 10.5% vs. 0.6% (*p* < 0.0001).

During the study period, a total of 4.2% (17/409) participants died. Five participants (1.2%) died before SVR and twelve (2.9%) during follow-up post-SVR.

### Pre-treatment evaluation at the NSP

All participants treated at the NSP (*n* = 162) underwent a pre-treatment evaluation, as depicted in Table 2. The distribution of HCV genotypes was 43.2%, 41.4% and 15.4% for genotype 3a, 1a/1b and 2, respectively. Mean fibrosis score was 7.5 kPa. The majority of participants (57.6%) had absent or mild fibrosis (F0–F1), while 10.8% had advanced fibrosis (F3) and 7.6% a fibrosis score > 12.5, indicating cirrhosis (F4). The overall mean APRI score was 0.51 (range 0.09–2.3).

**Table 2:** Pre-treatment evaluation among HCV treated at the Stockholm NSP (*n* = 162)

Genotype	*n* = 162 (%)
1a	62 (38.3)
1b	5 (3.1)
2	25 (15.4)
3a	70 (43.2)
Fibrosis score	*n* = 158
Mean (SD) kPa	7.5 (4.0)
Median (range) kPa	6.3 (3.4–33.8)
Fibrosis stages (kPa)	*n* = 158 (%)
F0-F1 (< 7)	91 (57.6)
F2 (7–9.4)	38 (24.1)
F3 (9.5–12.4)	17 (10.8)
F4 (≥ 12.5)	12 (7.6)
APRI score	*n* = 152
Mean (SD)	0.5 (0.4)
Median (range)	0.4 (0.09—2.3)
DAA treatment	*n* = 162 (%)
Glecaprevir + Pibrentasvir	64 (39.5)
Sofosbuvir + Ledispavir	61 (37.7)
Sofosbuvir + Velpatasvir	36 (22.2)
Elbasvir + Grazoprevir	1 (0.6)

### HCV treatment outcomes

**Fig. 1:**
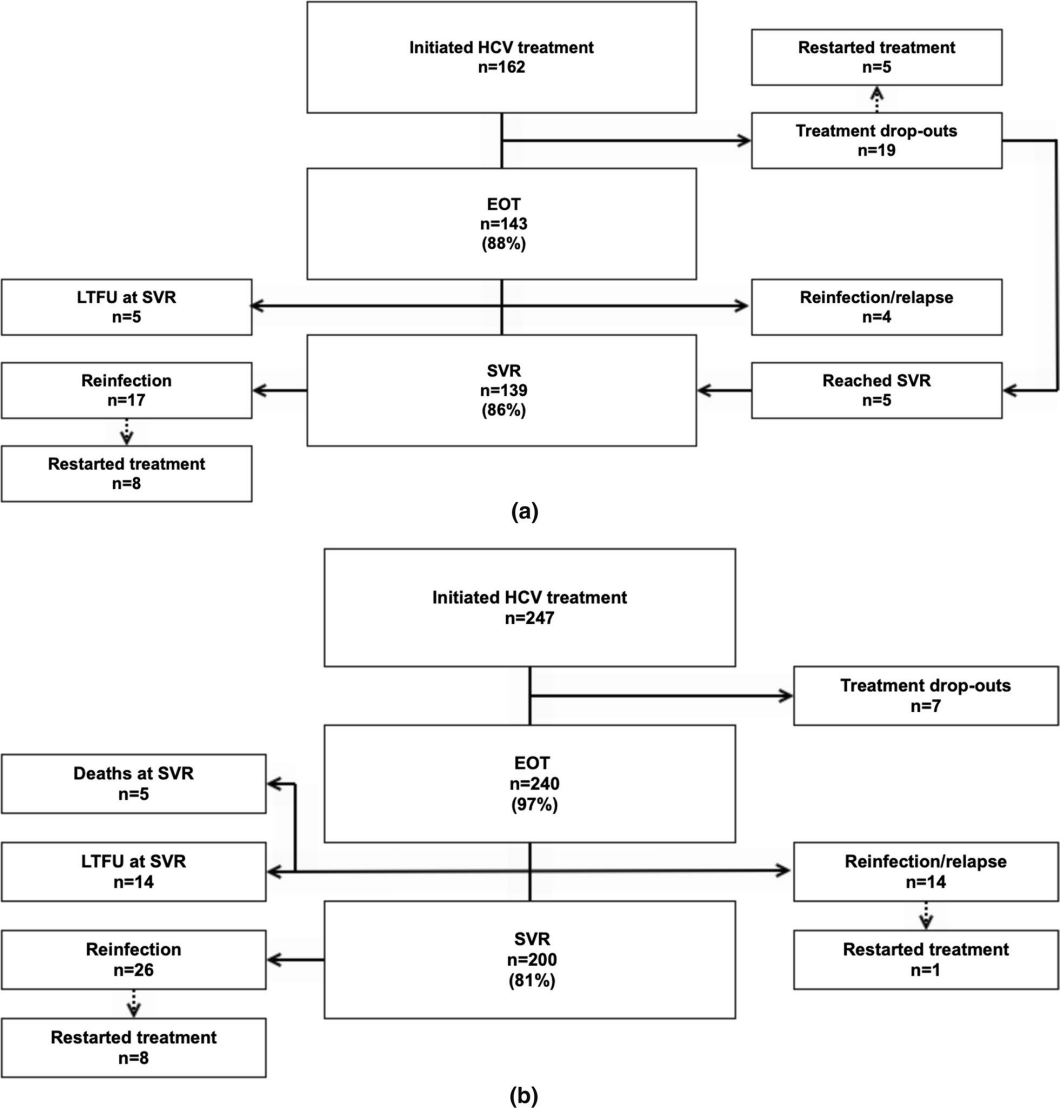
**a** HCV treatment flow chart for participants treated at the NSP. **b** HCV treatment flow chart for participants treated outside the NSP. *HCV* hepatitis C virus, *EOT* end of treatment, *SVR* sustained virological response, *LTFU* lost to follow-up

Among all HCV treated (*n* = 409), 93.6% (383/409) reached EOT and 82.9% (339/409) reached SVR. Treatment results and a flow chart for those treated at the NSP (*n* = 162) and outside the NSP (*n* = 247) are depicted in Fig. 1a, b. Of those treated at the NSP and outside the NSP, 88.3% and 97.2% (*p* < 0.001) reached EOT, and 85.8% and 81.0% (*p* = 0.23) reached SVR, respectively.

### Treatment dropouts and lost to follow-up

In total, there were 6.4% (26/409) treatment dropouts, with 11.7% (19/162) dropouts among participants treated at the NSP, compared to 2.8% among those treated elsewhere (*p* < 0.001). There were no demographic differences between the dropout groups. Among those treated at the NSP, the mean time in treatment before drop-out was 24 days (range 5–58) and for those still reaching SVR (*n* = 5), the mean number of missed days of DAA was 29 days (range 16–44). Stimulant use was more prevalent among dropouts compared to non-dropouts, 76.9% vs. 55.1% (*p* < 0.05). Consequently, opioid use and OAT were more prevalent among non-dropouts compared to dropouts, 39.9% vs. 11.5% (*p* < 0.05) and 32.4% vs. 7.7% (*p* < 0.01), respectively (data not shown).

Among the 49 participants that reached EOT but not SVR, 53.1% were LTFU at SVR, 36.7% had a relapse/reinfection between EOT and SVR, and 10.2% died. There were more participants LTFU among those who were treated outside the NSP compared to those treated at the NSP (*p* < 0.05), but there were no demographic differences between the two groups.

### Reinfection

A total of 339 participants were prospectively followed post-SVR (*n* = 139 treated at the NSP, and *n* = 200 treated outside the NSP). Participants were repeatedly tested every 3–6 months with a median of 84 days since last HCV test in the dataset (IQR 14–194 days). During follow-up there were a total of 43 HCV reinfections post-SVR, 17 among those treated at the NSP and 26 among those treated outside of the NSP, (*p* = 0.86). The mean time from SVR to reinfection was 291 days (range 34–954). Mean age at reinfection was 38.7 years, and 77.0% were men.

The overall HCV reinfection rate post-SVR was 9.3/100 PY (95% CI 7.0, 12.3), with follow-up time of 465 PY (mean individual follow-up time of 1.4 PY). The HCV reinfection rate among those treated at the NSP was 8.4/100 PY (95% CI 5.3, 13.2), with follow-up time 203 PY (mean follow-up time 1.5 PY) and among those treated outside the NSP 9.9/100 PY (95% CI 6.9, 14.3), with follow-up time 261 PY (mean follow-up time1.3 PY).

Factors associated with reinfection were younger age (*p* < 0.001), treatment while in prison (*p* < 0.01), homelessness (*p* < 0.05) and shorter duration of IDU (*p* < 0.0001), also corresponding to higher reinfection rates of 20.6, 65.8, 25.5 and 20.7/100 PY, respectively (Table 3).

**Table 3:** Reinfection and reinfection rates among participants who reached SVR (*n* = 339)

	No reinfection (*n* = 296)	Reinfection (*n* = 43)	*p* value	Reinfection rates (RR)	
				RR/100 PY	CI 95%
	*n* (%)	*n* (%)			
Gender	*n* = 296	*n* = 43			
Men	228 (77.0)	31 (72.1)	0.45	8.7	6.2–12.1
Women	68 (23.0)	12 (27.9)		11.5	6.8–19.6
Age	*n* = 296	*n* = 43			
Mean (SD)	46.2 (10.8)	38.7 (9.8)	< 0.0001		
Median (range)	48.7 (21–68)	36.0 (23–64)			
Age interval	*n* = 296	*n* = 43			
< 35	54 (18.2)	16 (37.2)	< 0.01	20.6	13.3–31.9
> 35	242 (81.8)	27 (62.8)		7.0	4.9–10.1
Country of birth	*n* = 292	*n* = 41			
Sweden	249 (85.3)	36 (87.8)	0.81	9.1	6.7–12.4
Outside of Sweden	43 (14.7)	5 (12.2)		8.4	3.6–19.5
Living situation	*n* = 249	*n* = 35			
House/apartment	155 (52.4)	13 (37.1)	< 0.01	4.8	2.9–8.2
Support housing	51 (17.2)	7 (20.0)	1.0	9.2	4.5–18.6
Treatment home	17 (5.7)	3 (8.6)	0.72	11.6	4.0–33.5
Prison	1 (0.3)	3 (8.6)	< 0.01	65.8	33.5–127.1
Homeless	25 (8.4)	9 (25.7)	< 0.05	25.5	14.6–44.9
Predominantly drug injected	*n* = 295	*n* = 43			
Stimulants/CS	173 (58.6)	22 (51.1)	0.41	7.6	5.1–11.3
Opioids/OP	109 (36.9)	18 (41.9)	0.61	11.5	7.4–17.7
Mixed CS/OP	12 (4.1)	3 (7.0)	0.42	19.8	7.2–54.7
Other	1 (0.3)	–	–	–	
Duration of IDU	*n* = 289	*n* = 43			
Mean (SD)	23.8 (13.1)	14.4 (10.1)	< 0.0001		
Median (range)	23.4 (1–52)	13.0 (1–44)			
Duration of IDU (interval)	*n* = 289	*n* = 43			
0–5	22 (7.6)	8 (18.6)	< 0.05	20.7	11.2–38.4
5+	267 (92.4)	35 (81.4)		8.4	6.1–11.6
OAT	*n* = 296	*n* = 43			
Yes	93 (31.4)	13 (30.2)	1.0	9.3	5.6–15.7
No	203 (68.6)	30 (69.8)		9.3	6.6–13.1
HIV	*n* = 296	*n* = 43			
Positive	17 (5.7)	2 (4.7)	1.0	9.0	2.4–33.7
Negative	279 (94.3)	41 (95.3)		9.3	7.0–12.5

## Discussion

In this study we report real-world HCV treatment baseline characteristics and treatment outcomes among PWID at the Stockholm NSP. Overall, there were 409 HCV treatments, of which 162 were initiated on site at the NSP and 247 treatments initiated outside the NSP. When comparing the two HCV-treated subgroups, participants treated at the NSP were older, predominantly injected stimulants, did not participate in OAT and were less likely to be HIV infected, compared to those treated outside of the NSP. These results indicate the need for a broad HCV treatment approach to reach different subgroups of PWID and that the NSP low-threshold setting specifically could benefit PWID not connected to other health care.

In our study setting, all participants were currently injecting drugs, but the cure rates were still high. Participants treated outside the NSP reached EOT to a higher extent, compared to those treated at the NSP, which was explained by less dropouts in that group. Notably, there were no differences regarding confirmed SVR, as participants treated outside the NSP instead were more likely to be LTFU between EOT and SVR.

Overall, 6.4% of participants were treatment dropouts, and among those treated at the NSP, 11.7% dropped out. This figure is in line with a Scottish study (11.6%), but higher than in a Norwegian study (4.7%), targeting PWID in a low-threshold program [45, 46]. The lower level of dropouts among those treated outside the NSP in our study could not be explained by demographic differences between the dropout groups. A possible explanation might instead be underreported non-successful treatments among those treated outside of the NSP or that treatment at ID clinics and OAT provided a greater incentive to continued healthcare contacts, but this needs to be investigated further.

As reported in other studies, we noted that recent stimulant injecting was associated with non-adherence, which on the other hand was not associated with lower levels of SVR [47, 48]. The median age was slightly higher among participants with HCV treated at the NSP, suggesting that the NSP reaches older PWID. However, this can also be explained by the higher mean age among amphetamine users compared to opioid users.

In a systemic review and meta-analysis of DAA treatment among OAT participants and PWID with recent drug use showed that treatment completion and SVR were 97.4% and 90.7% among OAT participants and 96.9% and 87.4% among PWID, respectively [21]. The somewhat lower SVR rates in these studies, compared to people not using drugs, could be explained by LTFU in these populations. This has raised the question whether SVR is the best indicator for cure among PWID, or rather, if cure could be synonymous with adherence and EOT, with a suggested focus on follow-up for possible reinfections [49]. However, LTFU has also been associated with more active and high-risk IDU, which suggests a higher risk of reinfection [29]. These studies correspond well to levels of EOT (93.6%) and SVR (82.9%) in our data, especially considering that participants in our cohort were PWID with current IDU, predominantly using stimulants, and with non-negligible levels of drop-out and LTFU. Recent studies also confirm setting-dependent levels of SVR with a high level of SVR in a low-threshold program in Norway (90%) and a lower level of SVR at a mobile HCV unit in Madrid (68%), indicating the need to tailor services for different populations and settings [45, 50].

The HCV incidence among PWID outside HCV treatment settings ranges from 5 to 40% per year, with a median incidence rate of 26/100 PY [2, 51–53]. Between 2013 and 2016, the overall incidence rate among non-treated PWID at the Stockholm NSP was 22/100 PY with a reinfection rate of 19/100 PY among those with previous HCV exposure [32]. In this study the overall reinfection rate post-SVR was 9.3/100 PY. A possible explanation for this lower reinfection rate could be that this subgroup of HCV-treated PWID constitutes a group with overall lower injection risk behaviors. Other explanations could be an effect of high-coverage NSP, decreasing levels of HCV prevalence among PWID, and more speculative, that HCV treatment itself may be a protective factor for reinfection, although this needs to be studied further.

In a meta-analysis on HCV reinfection after successful treatment among PWID, the reinfection rate was 6.2/100 PY (95% CI 4.3–9.0) among those who recently injected drugs and 3.8/100 PY among OAT participants [54]. Longer follow-up was associated with lower reinfection rate, indicating higher reinfection risk early post-SVR. In a recent Norwegian study, the reinfection rate was 3.7/100 PY (95% CI 1.6–7.4) among those with IDU during follow-up and 9.6/100 PY (95% CI 4.1–18.8) among those who reported mixed heroin/amphetamine injecting [45]. Other studies have reported higher levels of reinfection with reinfection rates of 14.8–18.9/100 PY among recent injectors and 19.9/100 PY among PWID treated at an NSP [46, 50, 55]. The highest reinfection rate in one study, 14.3/100 PY (11.1–18.5), was found among participants treated in prison, a trend which was also noted in our study [56]. Homeless and younger PWID also had higher reinfection rates in our study, indicating a need for further tailored interventions for these subgroups during HCV treatment and follow-up. In a modeling study, persistent treatment rates above 80/1000 (8%) among PWID resulted in an initial increased number of reinfections which then decreased with consistent HCV treatment over time [7]. This was explained by an increased pool of HCV-susceptible individuals after treatment-induced SVR. Thus, reinfections will occur and continuous surveillance and retreatment of reinfected PWID need to be a central part of the HCV elimination strategy [57].

A study by Rosenthal et al. describes the effectiveness of prescribing OAT in combination with HCV treatment among opioid users, with an SVR rate of 82% [58]. However, in an associated correspondence, Bach et al. call for strategies to also reach the increasing PWID population with stimulant use disorder in North America [59]. In Swedish NSP, over 60% of PWID predominantly use amphetamines, adding to the challenge of eliminating HCV as amphetamine use disorder, compared to OAT for opioid use disorder, lack effective pharmacotherapy treatment that retain patients in care [60, 61]. Instead, current treatment options for stimulant use are cognitive behavioral therapy, contingency management programs and psychosocial support. Amphetamine is also associated with high injection and sexual risk behaviors, and thus an increased risk for HCV transmission [62, 63]. Furthermore, a recent Swedish register study also noted a higher crude risk of liver-related death among HCV-infected amphetamine users compared to opioid users, highlighting the need to prioritize HCV treatment among PWID who use amphetamines [64]. In our study, we noted that the NSP is an important setting to treat HCV among people who use amphetamines, as other subgroups such as OAT patients are offered HCV treatment at OAT clinics and HIV patients are offered HCV treatment at ID clinics. To achieve HCV elimination targets, the need to engage people who predominantly inject amphetamine has been highlighted also in a study by Dibbs et al. [65].

Real-world data on HCV treatment and change in prevalence are now emerging. An Australian study examined treatment uptake and the viremic prevalence among PWID attending NSP nationally between 2015 and 2017 [66]. Within the sample population, treatment initiation increased from 10% in 2015 to 41% in 2017 and the HCV viremic prevalence declined from 43% in 2015 to 25% in 2017. In Tayside in Scotland, yearly uptake of treatment among PWID increased from 15 to 43% between 2014 and 2018, resulting in a decline in HCV viremia from 73 to 44% among HCV-antibody positive between 2010 and 2018 [67]. In line with this, cross-sectional data from the Stockholm NSP annual reports suggest that overall HCV prevalence has decreased from 52% in 2018 to 29% in 2021 among NSP participants as an effect of decreased injection risk behaviors and increased uptake of HCV treatment, but this warrants further studies [68, 69].

Lastly, the COVID-19 pandemic has resulted in delayed HCV treatments worldwide, which may result in increased HCV-related morbidity and mortality [70]. In Sweden, an overall 55% decrease in treatment initiations was noted during the first ten months of the pandemic [71]. A 30% decrease of HCV treatment initiations was also noted at the Stockholm NSP during the same period [72]. A recent modeling study, also taking the COVID-19 effects into account, concluded that Sweden is on-track to achieve three of the four WHO targets for HCV elimination. However, to reduce new infections, an increased access to harm reduction programs and a scale-up of HCV treatment among PWID is needed [71].

There are several limitations in our study. This was an observational study, at one NSP, which relied on routinely collected data and the results may not be generalizable to other settings. Also, as the majority of those with viral recurrence between EOT and SVR (where we were unable to distinguish viral relapse from reinfection) either had a negative HCV RNA at EOT or were considered compliant during treatment. This indicates a high possibility for cure, rather than relapse, which may have led to an underestimation of reinfections in this study. Furthermore, LTFU and our passive follow-up approach post-EOT and SVR may have resulted in both missed time of continuous negative HCV RNA, overestimating reinfection rates, or events of reinfections, underestimating reinfection rates. However, overall few participants were LTFU, which facilitated long-time follow-up and repeated HCV testing, constituting a major strength of this study. The NSP setting thus provides great opportunities for HCV treatment, follow-up and surveillance for PWID.

## Conclusion

In this study, we used real-world data to study HCV treatment outcomes among PWID at the Stockholm NSP. In this high HCV prevalence setting, with a majority of stimulant users, treatment success was high and the level of reinfections manageable. To reach HCV elimination, as proposed by WHO, there is a need to target specific PWID subgroups with HCV treatment, in both harm reduction and adjacent healthcare settings frequented by PWID.

## Data Availability

The datasets used by the study are available from the corresponding author on reasonable request.

## Abbreviations

APRI: Aspartate Aminotransferase (AST) to Platelet Ratio Index
CI: Confidence interval
COVID-19: Coronavirus disease 2019
EOT: End of treatment
DAA: Direct-acting antiviral
HIV: Human immunodeficiency virus
HBV: Hepatitis B
HCV: Hepatitis C
IDU: Injection drug use
IQR: Interquartile range
LSM: Liver stiffness measurement
LTFU: Lost to follow-up
NSP: Needle syringe programs
OAT: Opioid agonist treatment
PWID: People who inject drugs
PY: Person-years
RNA: Ribonucleic acid
SVR: Sustained virological response
WHO: World Health Organisation
